# PAR2-β-arrestin 2-ERK axis mediates *Malassezia globosa*-induced IL-17 response by disrupting ZO-1 in keratinocytes

**DOI:** 10.1016/j.isci.2026.115646

**Published:** 2026-04-08

**Authors:** Jinfeng Tang, Fanggu Li, Jiaqing Hong, Xueying Li, Ying Zhou, Jie Liu, Yiming Fan

**Affiliations:** 1 Clinical Research Center, Affiliated Hospital of Guangdong Medical University, Zhanjiang 524001, China; 2 Department of Dermatology, Affiliated Hospital of Guangdong Medical University, Zhanjiang 524001, China; 3 Experimental Animal Center, Affiliated Hospital of Guangdong Medical University, Zhanjiang 524001, China; 4 Dermatology, Plastic and Cosmetic Surgery Center, the First Dongguan Affiliated Hospital of Guangdong Medical University, Dongguan 523106, China

**Keywords:** Molecular biology, Immunology, Cell biology

## Abstract

*Malassezia*, a lipid-dependent commensal yeast of human skin, also functions as an opportunistic pathogen contributed to the pathogenesis of various skin and systemic diseases. Although the association between protease-activated receptor 2 (PAR2) and fungal infections has been partially characterized, its specific role in *Malassezia*-related dermatoses remains poorly elucidated. In this study, we demonstrated that PAR2 interacted with Zonula occludens-1 (ZO-1) and β-arrestin 2 to cooperatively regulate *M. globosa*-infected keratinocytes by Co-IP, PLA, and MbYTH. Utilizing *Malassezia* folliculitis patient samples, cellular infection models, and murine infection models, we demonstrated that *M. globosa* exploited the PAR2-β-arrestin 2-ERK signaling axis by disrupting ZO-1, consequently amplifying IL-17-mediated inflammatory responses. Notably, pharmacological inhibition or genetic ablation of PAR2 attenuated the *Malassezia*-induced IL-17 inflammation. Collectively, our results clarify PAR2 as a potential therapeutic target for *Malassezia*-infected diseases, while providing new insights into the pathogenic mechanisms of commensal fungi.

## Highlights

• Interaction among PAR2, ZO-1, and β-arrestin 2 modulates fungal infection
• PAR2-β-arrestin 2-ERK signaling axis disrupts ZO-1 in HaCaT cells
• PAR2-β-arrestin 2-ERK signaling axis amplifies IL-17 inflammatory responses
• PAR2 knockdown suppresses *Malassezia*-induced IL-17 inflammation

## Graphical abstract

**Figure:**
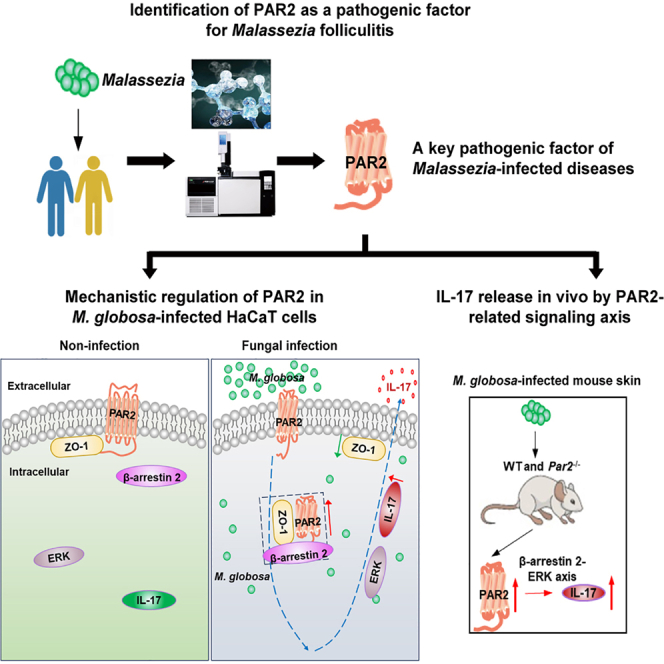

## Introduction

The genus *Malassezia globosa* (*M. globosa*) currently comprises 18 recognized species and is the most abundant symbiotic fungus on the skin of humans and homeothermic animals.^1^^,^^2^ As both commensals and opportunistic pathogens, they can cause several skin conditions including *Malassezia* folliculitis (MF), pityriasis versicolor, and seborrheic dermatitis, and aggravate atopic dermatitis, psoriasis, and even systemic diseases (such as respiratory, neurological, gastrointestinal, and genital infections).^3^^,^^4^^,^^5^ Among these species, *M. globosa* is the main pathogen causing MF, pityriasis versicolor, and psoriasis.^6^^,^^7^^,^^8^^,^^9^^,^^10^ Nevertheless, the precise pathogenic mechanisms underlying *M. globosa*-mediated diseases remain poorly understood. *Malassezia* is a lipid-dependent dimorphic yeast that lacks lipid metabolism genes, but it modifies the cutaneous microenvironment by secreting various hydrolytic enzymes such as protease, lipase, phospholipase, and sphingomyelinase, which disrupt the epidermal barrier and facilitate microbial colonization.^11^ These fungi primarily colonize the stratum corneum and hair follicle infundibula. Keratinocytes and immunocytes recognize *Malassezia* through pattern recognition receptors (PRRs), subsequently initiating inflammatory signaling pathways.^12^

Keratinocytes are major epidermal components that play a crucial role in maintaining the intact skin barrier and harmonious immune responses.^13^^,^^14^^,^^15^ As active innate immunocytes, keratinocytes express multiple PRRs that activate the microbial and host danger molecules and induce the release of inflammatory cytokines, chemokines, and antimicrobial peptides, thereby initiating various immune responses.^16^ *Malassezia* infection involves Th1, Th2, and Th17 immune responses.^1^^,^^17^ Previous studies found that *M. globosa* was the main *Malassezia* species in MF and promoted Th1 response of HaCaT cells through NLRP3 inflammasome.^9^^,^^17^ Emerging evidence suggests that *Malassezia* can trigger an IL-17-dependent inflammatory cascade in keratinocytes,^18^ contributing to the pathogenesis of some skin diseases including contact dermatitis, chronic pruritus, dust mites-induced atopic dermatitis, and *Staphylococcus aureus* infection.^19^^,^^20^^,^^21^^,^^22^^,^^23^ *Malassezia* can aggravate skin inflammation through Th17-biased immune response.^10^^,^^19^ Keratinocytes participate in this process via IL-36/MYD88 axis, which drives IL-17-dependent inflammation induced by *M. globosa*.^18^ In addition, *M. globosa* promotes IL-23 secretion of keratinocytes, which further amplifies pathogenic Th17 differentiation through TLR2/MYD88/NF-κB pathway.^24^ Although cumulative data confirm the association between *Malassezia* infection and IL-17 signaling in keratinocytes,^15^ the exact regulatory mechanism on this inflammatory response remains to be clarified. *Malassezia*-evoked Th17 immune response has a dual effect on the host, both antifungal and proinflammatory, possibly hinging on its symbiosis and pathogenicity.^25^^,^^26^ In mouse model of skin infection, *Malassezia* prevented fungal overgrowth through IL-23/IL-17 axis in intact skin but aggravated IL-23/IL-17 axis-dependent skin inflammation in damaged skin.^19^ In atopic dermatitis and psoriasis, *Malassezia* can promote keratinocyte proliferation and exacerbate skin inflammation via triggering Th17 immune response.^27^ Furthermore, the fungal infection (especially MF) risk increased following administration of IL-17 inhibitors, implying that IL-17 is crucial for antifungal immunity.^28^

Protease-activated receptor 2 (PAR2) belongs to the rhodopsin G protein-coupled receptors (GPCRs) and is widely expressed in epithelial/endothelial cells, platelets, smooth muscle cells, nerve cells, and immunocytes.^29^^,^^30^^,^^31^^,^^32^ PAR2 is primarily activated by serine proteases and plays a significant role in the pathogenesis of coagulation, cardiovascular diseases, cancer, and inflammatory skin diseases.^33^ During PAR2 activation, its N-terminal is firstly cleaved by proteases to expose a new N-terminal self-activating tethered ligand (TL), followed by molecular docking with transmembrane receptors, resulting in intracellular signal transduction.^32^ PAR2 can act as a non-classical PRR, exerting immunoregulatory role in various fungal infections.^34^^,^^35^^,^^36^ *Aspergillus fumigatus* and *Alternaria* can promote IL-6, TNF-α, and GM-cerebrospinal fluid (CSF) secretion in airway epithelium via PAR2, inducing airway epithelial inflammation and allergic asthma.^34^^,^^36^ *Alternaria* serine protease can not only rapidly release IL-33 by activating PAR2, but also activate PAR2/β-arrestins pathway to induce Th2 immune response, thereby aggravating airway inflammatory response.^37^ *Candida albicans* Sap6 activated PAR2 through RGD (RGDRGD integrin-binding motifs) domain to initiate release of IL-1β and IL-8 in human oral epithelial cells;^38^ *Paracoccidioides brasiliensis* induced secretion of IL-6 and IL-8 in lung epithelial cells A549 by activating PAR2.^35^ At present, the role of PAR2 in fungal infections mainly focuses on Th1 and Th2 immune responses, with little attention to Th17 immune responses. It is unknown whether PAR2 is involved in the pathogenicity of *Malassezia* infection.

In this study, we systematically investigated the effect of PAR2 on IL-17-driven inflammatory response in *Malassezia*-infected keratinocytes and mice.

## Results

### Expression of PAR2, IL-17, and TJ proteins in MF

**Figure 1:**
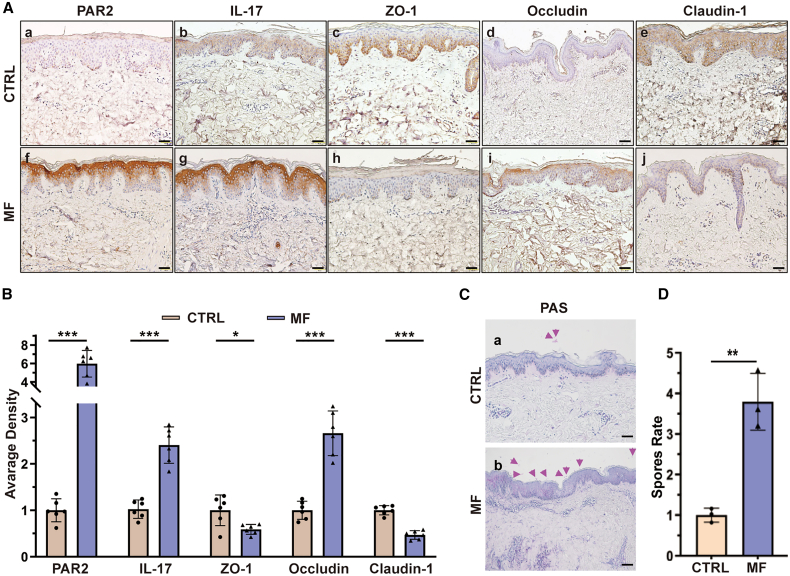
Expression of PAR2, IL-17, and TJ proteins in *Malassezia* folliculitis (A and B) PAR2, IL-17, ZO-1, occludin, and claudin-1 were validated in MF and CTRL skin tissue by immunohistochemical staining (IHC). Values are mean ± SD of six samples from two independent experiments. Statistical analysis was performed using a two-way ANOVA with Šídák’s multiple comparisons test (∗*p* < 0.05; ∗∗∗*p* < 0.001). (C and D) The spores rate of CTRL and MF groups by Periodic Acid-Schif staining. Purple arrows indicate the spores. Scale bars, 50 μm. Data are presented as mean ± SD of triplicate wells from three independent experiments. Statistical significance was determined using unpaired two-tailed Student’s *t* tests (∗∗*p* < 0.01).

To elucidate the underlying mechanism of MF, we performed liquid chromatography-tandem mass spectrometry (LC-MS/MS) analysis on three paired MF lesions and normal skin samples, followed by comprehensive bioinformatics analysis to identify differentially expressed proteins (DEPs). Proteomic profiling revealed significant enrichment of DEPs in several pathways, including complement and coagulation cascades, cell adhesion, extracellular matrix (ECM) receptor interactions, cytokines signaling, and viral infection responses (Figures S2A–S2F). The most prominent DEPs were PAR2, IL-17, and key tight junction (TJ) proteins (Zonula occludens [ZO-1], occludin, claudin-1), which were confirmed by immunohistochemistry. Compared with normal skin (CTRL), PAR2, IL-17, and occludin expression was increased, while ZO-1 and claudin-1 expression was decreased in six MF lesions (Figures 1A and 1B). Therefore, these results suggest that PAR2, IL-17, and TJ dysregulation may contribute to the pathogenesis of MF. Furthermore, periodic acid-Schiff (PAS) staining revealed that skin-colonizing spores were more abundant in MF than in normal skin (Figures 1C and 1D).

### Impact of *M. globosa* infection on PAR2 activation, IL-17 production, and TJ integrity in HaCaT cells

**Figure 2:**
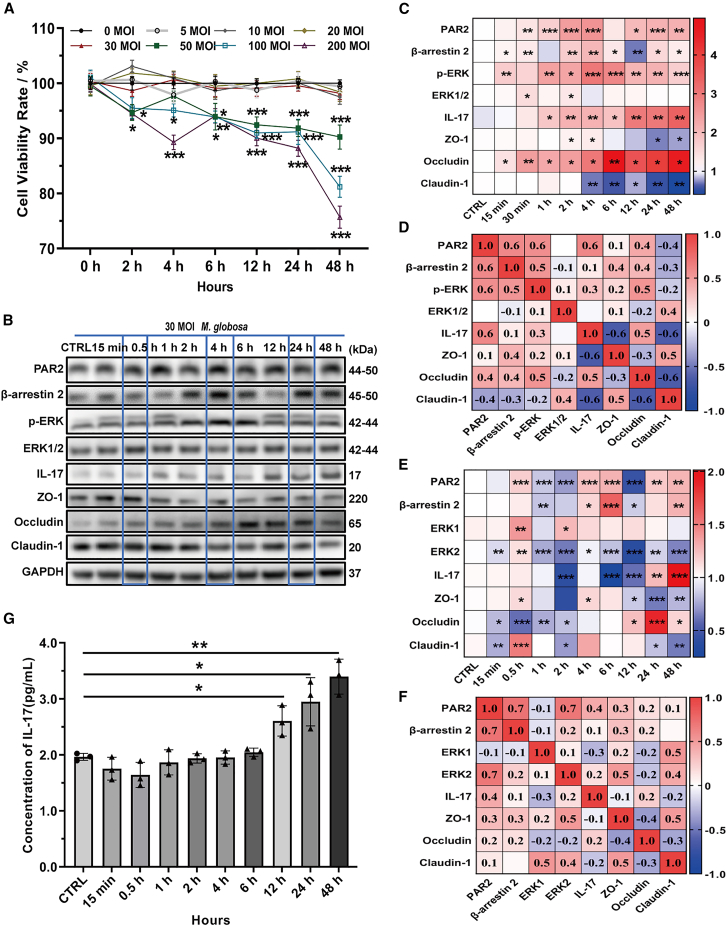
Impact of *M. globosa* infection on PAR2 activation, IL-17 production, and TJ integrity in HaCaT cells (A) Cell survival rate was detected by CCK8 assay. (B–D) Protein expression and correlation analysis in HaCaT cells infected with 30 MOI *M. globosa* was evaluated by western blot at various time. (E and F) mRNA levels and correlation analysis were measured by reverse transcription quantitative PCR (RT-qPCR). Red indicated upregulated gene expression, green indicated downregulated gene expression. (G) IL-17A levels were detected by ELISA test in the supernatant of HaCaT cells infected with 30 MOI *M. globosa*. Red indicated upregulated gene expression (C and E) or positive correlation (D and F), blue indicated downregulated gene expression (C and E) or negative correlation (D and F).These experiments (A, B, E, and G) were representative of 3 independent experiments. Values are means ± SD. a two-way ANOVA with Bonferroni’s (A), or Dunnet’s (G) multiple comparisons test was applied. ∗*p* < 0.05, ∗∗*p* < 0.01, ∗∗∗*p* < 0.001. Correlation analysis is performed using Pearson’s correlation coefficient analysis. See also Figure S2.

*M. globosa*, the predominant pathogen in MF,^9^ elicits diverse proinflammatory responses in keratinocytes.^39^ Emerging evidence suggests that keratinocytes orchestrate *Malassezia*-induced, IL-17-mediated cutaneous inflammation via IL-36 and IL-23 signaling.^18^^,^^24^ To delineate the functional consequences of *M. globosa* infection on keratinocyte, we first evaluated cellular viability under varying multiplicities of infection (MOI). CCK-8 assays revealed a significant dose-dependent reduction in HaCaT cell viability at 50–200 MOI, whereas 0–30 MOI exhibited no detectable cytotoxicity (Figure 2A). Consequently, 30 MOI was chosen for subsequent experiments to maximize infection efficiency and preserve cellular homeostasis.

PAR2 has been implicated in the regulation of IL-17 across multiple infections and inflammatory contexts, including *Helicobacter pylori* and *Porphyromonas gingivalis* infections, as well as chronic pain disorders.^40^^,^^41^^,^^42^ We therefore postulated that *M. globosa* might similarly engage PAR2-dependent pathways in keratinocytes. Notably, PAR2 is known to modulate TJ dynamics in keratinocytes and pulmonary/intestinal epithelial cells through β-arrestin-mediated ERK1/2 phosphorylation, influencing both barrier integrity and inflammatory responses.^43^^,^^44^^,^^45^^,^^46^ To investigate this mechanism, we performed temporal profiling of PAR2, β-arrestin 2, phospho-ERK (*p*-ERK), IL-17, and TJ proteins (ZO-1, occludin, and claudin-1) in *M. globosa-*infected HaCaT cells. Western blotting and reverse transcription quantitative PCR (RT-qPCR) assays revealed significant temporal modulation of these markers at 0.5, 4, and 24 h post-infection (Figures 2B–2F).

Furthermore, ELISA revealed a significant increase in IL-17 secretion 12 h post-infection (Figure 2G), indicating a time-dependent induction of IL-17 by *M. globosa* at 30 MOI. The peak IL-17 response observed at 24 h post-infection paralleled our previous findings on *M. globosa*-driven Th1-polarized immunity,^17^ further highlighting the yeast’s ability to skew keratinocyte-mediated inflammatory networks.

Collectively, these data demonstrate that *M. globosa* infection may potentiate PAR2 signaling, compromise TJ integrity, and enhance IL-17 release in keratinocytes, thereby fostering a proinflammatory microenvironment conductive to MF pathogenesis.

### Ultrastructural characterization of HaCaT cells and *M. globosa* interaction during infection

**Figure 3:**
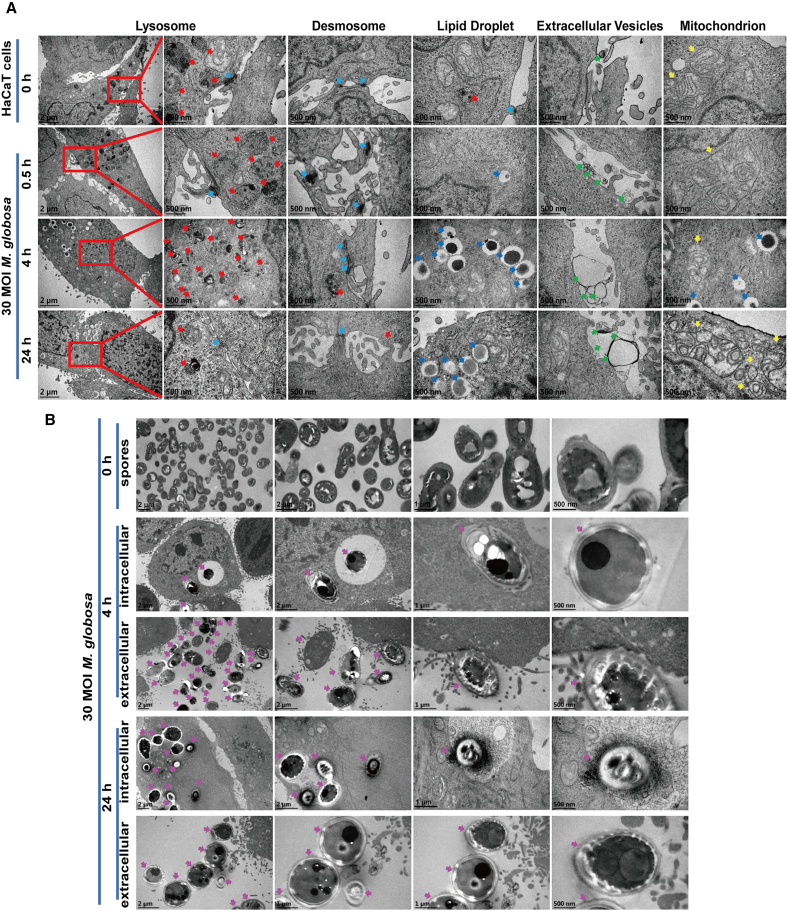
Ultrastructural alterations of HaCaT cells and *M. globosa* interaction during infection (A) Ultrastructural alterations of cells. Red arrows denote lysosomes or autophagolysosomes, light blue arrows denote desmosome-like structures, dark blue arrows denote lipid droplets, green arrows denote extracellular vesicles, and yellow arrows denote mitochondria. (B) Ultrastructural changes of intracellular and extracellular spores. Purple arrows indicate intracellular spores. Scale bars, 2, 1, and 500 nm. Three biological replicates were conducted. Representative data are presented.

*M. globosa* typically maintains skin homeostasis through complex microenvironmental interactions,^47^ but can transition into a pathogenic state under cutaneous dysbiosis, leading to *Malassezia*-associated dermatoses.^24^ To characterize the cytopathological effects of this transition, transmission electron microscopy (TEM) was performed on HaCaT cells infected with 30 MOI *M. globosa* at 0, 0.5, 4, and 24 h post-infection. Infected keratinocytes exhibited distinct time-dependent morphological alterations as follows: (1) desmosome formation and extracellular vesicles were dramatically escalatory at 0.5 h, suggestive of the initiation of immediate cellular defense; (2) secondary lysosomes and electron-dense lipid droplets were prominent at 4 h, indicative of active metabolic and phagocytic responses; and (3) mitochondrial structures were severely damaged at 24 h, such as complete disintegration of the outer membrane, dissolution of inner cristae into monolayered annular structures, and dispersion of inner membrane remnants in the cytoplasm (Figure 3A).

Concurrently, some *M. globosa* spores entered HaCaT cells at 4 h post-infection. Intracellular spores exhibited nuclear chromatin condensation and increased electron density; intravacuolar spores remained spherical with attenuated cell wall staining; and extracellular spores were polymorphic, with some partially engulfed by keratinocyte pseudopodia through undulated electron-dense contact zones. At 24 h, intracellular spores underwent further degradation, releasing fine electron-dense granules, some of which escaped from necrotic keratinocytes. However, extracellular spores retained a morphology similar to that of intravacuolar spores at 4 h (Figure 3B). Collectively, these ultrastructural alterations were progressive and conspicuous at 24 h post-infection, suggesting the underlying interaction between *M. globosa* and keratinocytes during infection.

### Activation of PAR2-β-arrestin 2-ERK signaling axis and IL-17 in *M. globosa*-infected HaCaT cells

The PAR2 signaling pathway has been shown to function through a β-arrestin-mediated mechanism that is independent of G protein coupling.^48^^,^^49^ The β-arrestin acts as a critical regulator, not only terminating G protein signaling but also facilitating PAR2 internalization and scaffolding the formation of the PARs-β-arrestin-Raf-1-MEK-1-ERK1/2 complex. This process prolongs cytosolic ERK1/2 activity by delaying its nuclear translocation.^50^ Given PAR2’s role in IL-17 regulation across various diseases,^51^^,^^52^ we investigated its involvement in *M. globosa*-induced IL-17 production in HaCaT cells. Our temporal analysis revealed that *M. globosa* infection significantly triggered IL-17 release at 12 h post-infection. Western blotting and RT-qPCR correlation analysis indicated that IL-17 production was mediated through PAR2 activation, with potential involvement of β-arrestin 2, ERK2, and TJ protein ZO-1 (Figures 2B–2F).

**Figure 4:**
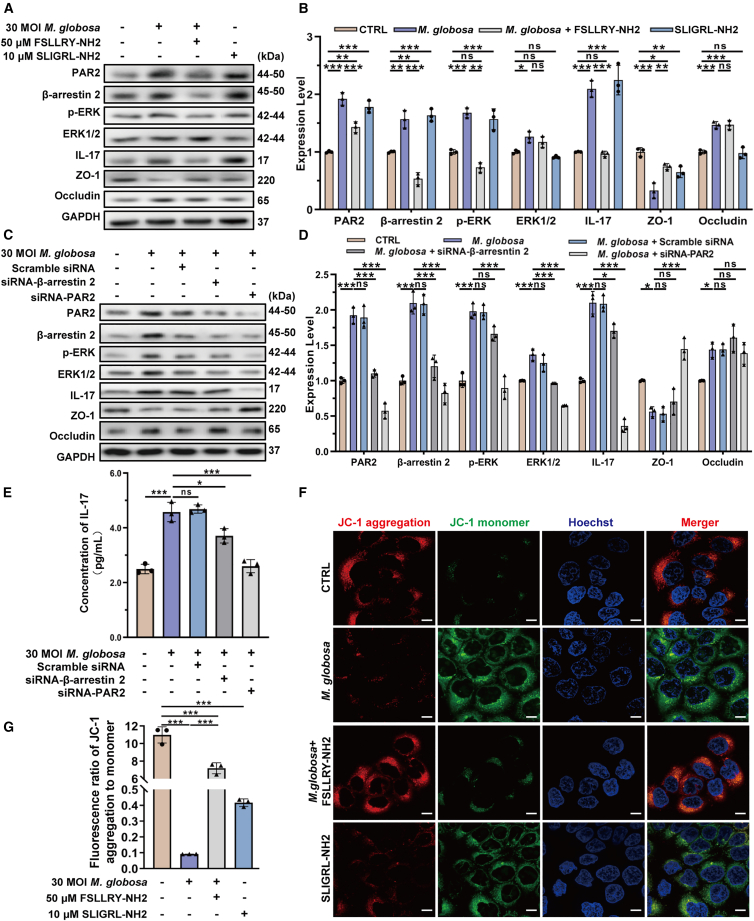
Activation of PAR2-β-arrestin 2-ERK signaling axis and IL-17 in *M. globosa*-infected HaCaT cells (A and B) Protein expression level following PAR2 antagonist intervention using western blotting. (C and D) PAR2-related pathway proteins expression following siRNA transfection by western blotting. (E) IL-17 levels in cell supernatants after siRNA transfection by ELISA. (F and G) Fluorescence of JC-1 aggregates to monomers. Scale bars, 10 μm. These experiments (A, C, E, and F) were representative of 3 independent experiments. Values are mean ± SD. Statistical analysis was performed using a two-way ANOVA with Šídák’s multiple comparisons test (ns, no statistical significance; ∗*p* < 0.05; ∗∗*p* < 0.01; ∗∗∗*p* < 0.001). See also Figure S3.

Based on previous findings that PAR2 enhances intestinal barrier function through β-arrestin-ERK1/2-dependent upregulation of TJs,^45^ we employed pharmacological and genetic approaches to delineate this pathway in keratinocytes. HaCaT cells were treated with 50 μM PAR2 antagonist FSLLRY-NH2^53^^,^^54^ for 1 h (Figure S3), followed by infection with 30 MOI *M. globosa* at 24 h. Concurrently, HaCaT cells were treated with 10 μM PAR2 agonist SLIGRL-NH2 for 24 h. The PAR2 antagonist reversed *M. globosa*-induced upregulation of β-arrestin 2, phospho-ERK, IL-17, and occludin, as well as the downregulation of ZO-1 in HaCaT cells (Figures 4A and 4B). PAR2-specific small interfering RNA (siRNA) abolished *M. globosa-*induced increases in β-arrestin 2, *p*-ERK, and IL-17, while β-arrestin 2 siRNA blocked the elevation of *p*-ERK and IL-17. Both the treatments normalized ZO-1 and IL-17 expression (Figures 4C–4E).

Notably, although *M. globosa* infection caused significant mitochondrial damages of HaCaT cells, including outer membrane disintegration and cristae dissolution, the PAR2 antagonist FSLLRY-NH2 effectively prevented *M. globosa*-induced loss of mitochondrial membrane potential (Figures 4F and 4G). These collective findings indicate that PAR2-β-arrestin 2-ERK axis may be pivotal in *M. globosa*-induced IL-17 release in HaCaT cells.

### PAR2 interacts with β-arrestin 2 and ZO-1 in *M. globosa*-infected HaCaT cells

**Figure 5:**
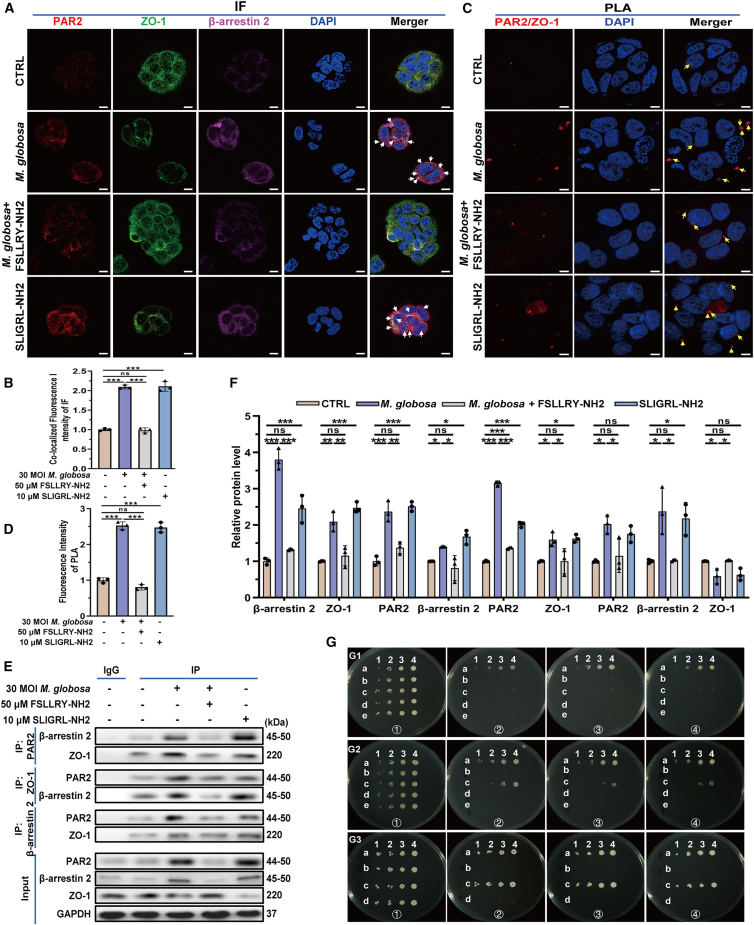
PAR2 interacts with β-arrestin 2 and ZO-1 in *M. globosa*-infected HaCaT cells (A and B) Expression and co-localization of PAR2, β-arrestin 2, and ZO-1 in HaCaT cells by immunofluorescence. White arrows: co-localization. Scale bars, 10 μm. (C and D) Expression and co-localization of PAR2 and ZO-1 by PLA. The yellow arrows indicated the PLA-positive signal, Scale bars, 20 μm. (E and F) Interaction regulation of PAR2, β-arrestin 2, and ZO-1 using CoIP. These experiments (A, C, and E) were representative of three independent experiments. Data are presented as mean ± SD. Statistical analysis was performed using a two-way ANOVA with Šídák’s multiple comparisons test (ns, no statistical significance; ∗*p* < 0.05; ∗∗*p* < 0.01; ∗∗∗*p* < 0.001). (G) PAR2 interaction with ZO-1 and β-arrestin 2 using MbYTH. G1. MbYTH of PAR2 and control NR1I2. (a) Positive control, (b) negative control, (c) experimental groups: pDHB1-PAR2 and pPR3-N-NR1I2, (d) autoactivation 1: pDHB1-PAR2, and (e) autoactivation 2: pPR3-N-NR1I2. G2. MbYTH of PAR2 and ZO-1. (a) Positive control, (b) negative control, (c) experimental groups: pDHB1-PAR2 and pPR3-N-ZO-1, (d) autoactivation 1: pDHB1-PAR2, and (e) autoactivation 2: pPR3-N-ZO-1. G3. MbYTH of PAR2 and β-arrestin 2. (a) Positive control, (b) negative control, (c) experimental groups: pDHB1-PAR2 and pPR3-N-β-arrestin 2, (d) autoactivation: pPR3-N-β-arrestin 2. SD/-Leu/-Trp medium; SD/-Leu/-Trp/-His medium containing 5 mM 3-AT; SD/-Leu/-Trp/-His/-Ade medium; SD/-Leu/-Trp/-His/-Ade medium containing 5 mM 3-AT. 1/2/3/4 were 10,000/1,000/100/10 times dilution of the original bacterial solution.

To elucidate the functional interplay between PAR2, β-arrestin 2, and ZO-1 during *M. globosa* infection, HaCaT cells were pretreated with the PAR2 antagonist FSLLRY-NH2 (50 μM) for 1 h, followed by infection with *M. globosa* (30 MOI) for 24 h. Immunofluorescence and proximity ligation assay (PLA) tests revealed enhancive co-localizations of PAR2 with both β-arrestin 2 and ZO-1 upon *M. globosa* infection or stimulation with PAR2 agonist SLIGRL-NH2 (Figures 5A–5D). Co-immunoprecipitation (Co-IP) assays further confirmed that *M. globosa* infection and PAR2 agonist pretreatment significantly strengthened the physical interaction among PAR2, ZO-1, and β-arrestin 2, which was effectively attenuated by PAR2 antagonist (Figures 5E and 5F).

To independently validate these protein interactions, full-length coding sequences of PAR2, ZO-1, and β-arrestin 2 were cloned into plasmid vectors and expressed in the yeast two-hybrid strain NMY51. Screening results identified molecular interactions among PAR2, ZO-1, and β-arrestin 2 (Figure 5G), supporting the role of PAR2 as a scaffold protein that facilitates complex formation in response to *M. globosa* challenge.

### Attenuation of PAR2-β-arrestin 2-ERK signaling and IL-17 responses in *Par2*^−/−^ mice with skin *M. globosa* infection

To evaluate the role of the PAR2-β-arrestin 2-ERK signaling axis and IL-17 in *Malassezia* infection *in vivo*, we established a cutaneous *M. globosa* infection model in mice, as previously described.^17^^,^^24^ This model was designed to delineate the mechanism by which *M. globosa* induces IL-17 production in keratinocytes via the PAR2-β-arrestin 2-ERK signaling axis. Twelve *Par2*^−/−^ or wild-type (WT) mice were randomly assigned to vehicle and *M. globosa* groups. Animals were euthanized on day 7 post-infection.^17^

**Figure 6:**
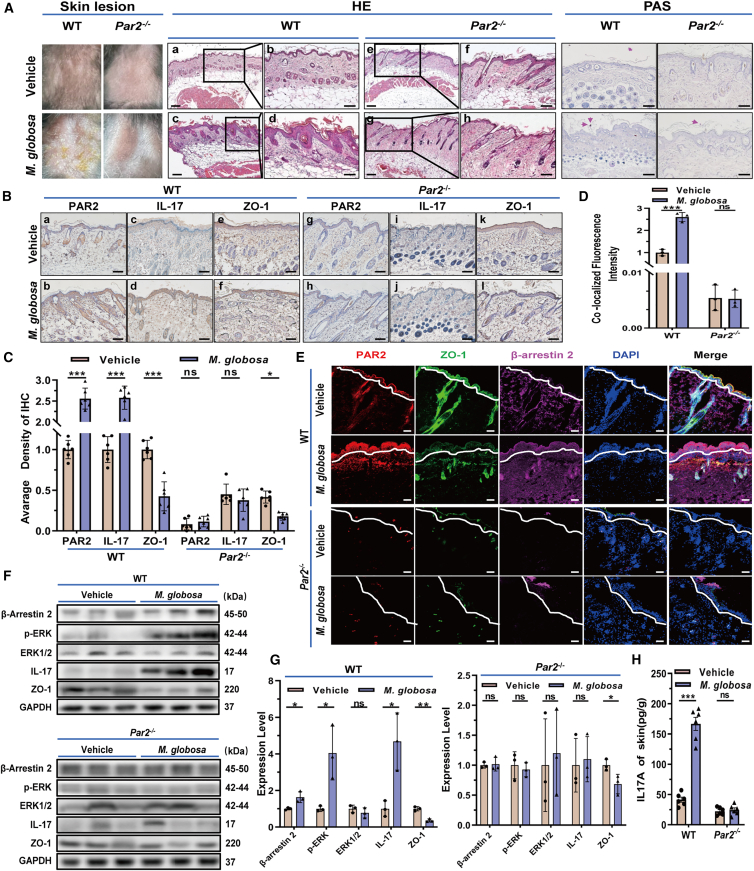
Attenuation of PAR2-β-arrestin 2-ERK signaling and IL-17 responses in *Par2*^*-/-*^ mice with skin *M. globosa* infection (A) Skin lesions and pathological staining of mouse skin. Scale bars, 50 μm. (B and C) Expression level of PAR2, IL-17, ZO-1 by IHC. (D and E) Expression and co-localization of PAR2, β-arrestin 2, and ZO-1 by immunofluorescence. White dotted line indicated the basement membrane zone. Scale bars, 50 μm. (F and G) Expression of related proteins using western blotting. (H) IL-17 levels using ELISA. (B, C, and H) For each group, *n* = 6. These experiments (A, D, E, F, and G) were representative of 3 independent experiments. Data are presented as mean ± SD. Statistical significance was determined using a two-way ANOVA with Šídák’s multiple comparisons test (ns, no statistical significance; ∗*p* < 0.05; ∗∗*p* < 0.01; ∗∗∗*p* < 0.001).

In WT mice, infection with *M. globosa* induced remarkable clinical and histopathological changes by day 7, including scaling, crusting, epidermal hyperplasia, spongiosis, and dermal vascular dilation with lymphohistiocytic infiltration (Figure 6A). In contrast, these clinicopathological manifestations were markedly attenuated in *Par2*^−/−^ mice. Immunohistochemical staining (IHC) results showed that the expression trends of PAR2, IL-17, and ZO-1 in WT mice infected with *M. globosa* were similar to those observed in MF, whereas *Par2*^−/−^ mice were not affected by *M. globosa* infection. These findings suggest that PAR2 plays a crucial regulatory role in the pathogenesis of MF (Figures 6B and 6C).

Immunofluorescence analysis revealed prominent co-localization of PAR2 with β-arrestin 2 and ZO-1 in WT mice following infection, which was absent in *Par2*^−/−^ mice (Figures 6D and 6E). Furthermore, infection-induced changes in the expression and distribution of PAR2, β-arrestin 2, and ZO-1 were evident in WT mice but were negligible in *Par2*^−/−^ mutants. Western blotting found that *M. globosa* infection upregulated PAR2, β-arrestin 2, *p*-ERK, and IL-17 expression in WT mice but not in *Par2*^−/−^ mice. Downexpression of ZO-1 was observed in both genotypes, particularly in WT mice (Figures 6F and 6G). ELISA results indicated that cutaneous IL-17 levels was elevated in *M. globosa*-infected WT mice and minimal in *Par2*^−/−^ mice (Figure 6H).

Collectively, these *in vivo* results substantiate the involvement of the PAR2-β-arrestin 2-ERK axis in *M. globosa-*induced cutaneous inflammation and IL-17 production.

## Discussion

Previous studies have established the role of Th17 immunity in *Malassezia* infection and other fungal infections of mouse skin.^18^^,^^19^^,^^24^^,^^25^^,^^55^ However, the mechanism by which *Malassezia* induces an IL-17 response in keratinocytes remains unclear. As a major component of the epidermis, keratinocytes not only provide a physical barrier but also initiate immune responses by recruiting and activating immune cells, thereby triggering local inflammation.^56^^,^^57^ *Malassezia* infection triggers the coordinated antifungal immunity mediated by the IL-17 signaling pathway in keratinocytes, which promotes cell migration and exacerbates cutaneous inflammation.^15^^,^^18^^,^^19^^,^^24^ Our results demonstrate the presence of an IL-17 immune response in MF lesions, *M. globosa*-infected HaCaT cells, and mouse skin, consistent with previous studies.

The proteases secreted by *M. globosa* function as major virulence factors, promoting nutrient acquisition through proteolysis and degradation of host tissues, facilitating adhesion by modifying host cells, regulating immune responses via secretion of proinflammatory cytokines, and being directly recognized by host membrane-bound PRRs.^58^^,^^59^^,^^60^^,^^61^^,^^62^^,^^63^ This study identified that PAR2, a non-canonical PRR, exhibited time-dependent upregulation during the infection process in *M. globosa*-infected HaCaT cells and was positively correlated with IL-17 levels. Serine protease is the primary protease secreted by *M. globosa* and acts as a key activator of PAR2.^9^^,^^64^ The intracellular endocytosis of PAR2 depends on its interaction with β-arrestin, which is crucial for the targeted regulation of ERK1/2.^50^^,^^65^ Inhibition of the PAR2-β-arrestin-ERK1/2 axis has been shown to overcome osimertinib resistance in non-small cell lung cancer,^66^ and this signaling pathway can also regulate intestinal inflammation by modulating mucosal barrier integrity through TJ proteins of epithelial cells.^46^ Although PAR2 mediates various physiological and pathological processes via the β-arrestin 2-ERK axis, its role in *M. globosa* infection remains unreported. In both cellular and mouse infection models, we observed that PAR2 inhibition or knockout restored the downregulation of TJ proteins, upregulation of β-arrestin 2-ERK1/2 axis, and IL-17-driven inflammatory response in *M. globosa*-infected keratinocytes. Further studies revealed that PAR2 interacted with β-arrestin 2 and ZO-1 to regulate the inflammatory response. Interestingly, we found that ZO-1, occludin, and claudin-1 were all downregulated in MF, but only ZO-1 was restored by PAR2 intervention, indicating that PAR2 regulates ZO-1 expression and interacts with ZO-1 to modulate the infection process. This differential regulation may be related to the structural and functional characteristics of ZO-1, occludin, and claudin-1. PAR2-regulated TJs are believed to participate in the pathogenesis of chronic inflammatory diseases. For instance, PAR2 downregulates ZO-1 and claudin-1 expression, contributing to the epithelial barrier dysfunction in allergic rhinitis.^67^ TJs are composed of transmembrane proteins and plaque proteins: transmembrane proteins include claudins, TJ-related MARVEL proteins (such as occludin, tricellulin, and MarvelD3), and junction adhesion molecules (JAM); plaque proteins include cingulin (ZO) 1–3, MUPP-1 and cingulin.^68^ PAR2 activation leads to TJ disruption in epithelial cells and increases barrier permeability through p38 mitogen-activated protein kinase (MAPK) activation.^69^ In contrast, treatment with *Pseudomonas aeruginosa* elastase temporarily disrupts the epithelial barrier, downregulated transmembrane proteins claudin-1 and -4, occludin, and tricellulin, but does not downregulate scaffold proteins ZO-1 and -2 or adhesion junction proteins E-cadherin and β-catenin.^70^ Our results indicate that PAR2 regulates the scaffold protein ZO-1 in *Malassezia*-infected keratinocytes, while downregulation of membrane proteins occludin and claudin-1 may be directly related to the destructive effect of *Malassezia* proteases. Additionally, using TEM and JC-1 staining, we first demonstrated that *M. globosa* infection disrupts the mitochondrial structure and membrane potential of HaCaT cells, an effect reversed by PAR2 inhibitor. The PAR2 inhibitor AZ3451 can significantly improve mitochondrial function by increasing intracellular adenosine triphosphate (ATP) levels, restoring mitochondrial membrane potential, and reducing mitochondrial reactive oxygen species (ROS).^71^^,^^72^^,^^73^ The specific mechanism by which PAR2 regulates mitochondria function in *Malassezia*-infected HaCaT cells requires further investigation.

**Figure 7:**
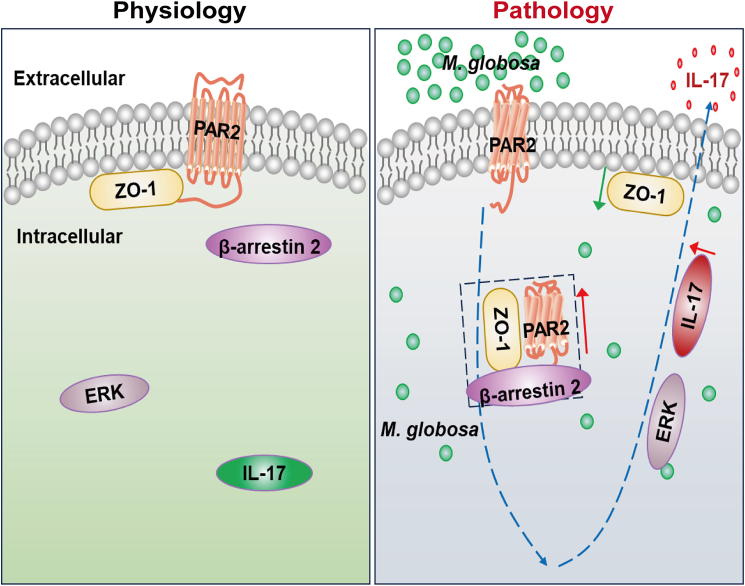
The possible mechanism of PAR2-β-arrestin 2-ERK signaling pathway on IL-17 release in keratinocytes On physiological state, PAR2, β-arrestin 2, and ZO-1 are in resting state, and ZO-1 is crucial for skin barrier maintenance. After overgrown *M. globosa* invades keratinocytes, the interaction of PAR2 with β-arrestin 2 and ZO-1 is enhanced and internalized, which upregulates ERK1/2 expression and ZO-1 disruption, thus promoting intracellular expression and extracellular release of IL-17.

In conclusion, this study demonstrates that PAR2 disrupts ZO-1 to mediate IL-17-driven inflammatory response in *M. globosa*-infected HaCaT cells and mouse skin via activation of the β-arrestin 2-ERK signaling pathway (Figure 7). Thus, PAR2 participates in the pathogenesis of *Malassezia*-related skin diseases and may represent a promising therapeutic target.

### Limitations of the study

In this study, we demonstrated that *M. globosa* activated the PAR2-β-arrestin 2-ERK signaling pathway in HaCaT cells and mouse skin, thereby mediating IL-17-driven inflammatory response. However, this effect remains to be testified using NHEK and skin organoids. Meanwhile, the specific functional interaction regions and mechanisms of PAR2/β-arrestin 2/ZO-1 need to be verified. In addition, the role of PAR2 in the transition between commensal and pathogenic *Malassezia* is well worth investigating.

## Resource availability

### Lead contact

Further information and requests for resources and reagents should be directed to and will be fulfilled by the lead contact, Yiming Fan (ymfan1963@163.com).

### Materials availability

Materials used in the study are commercially available. This study did not generate new unique reagents.

### Data and code availability

• All data reported in this paper will be shared by the lead contact upon reasonable request.
• Proteomics data of MF have been deposited at Science Data Bank and are publicly available as of the date of publication at Science Data Bank: https://doi.org/10.57760/sciencedb.29934 (listed in the key resources table). Raw data have been deposited at Mendeley Data: https://data.mendeley.com/datasets/ffxppsr7p2/1.
• No new code was generated during the course of this study.
• Any addition information required to reanalyze the data reported in this paper is available from the lead contact upon reasonable request.

## Acknowledgments

We thank Nicola Lopizzo for scientific editing. We thank applied protein technology and Shenzhe Biotech for excellent technical support. This work was supported by the 10.13039/501100001809National Natural Science Foundation of China (grant nos. 81673071 and 82404162).

## Author contributions

Conceptualization, Y.F. and J.T.; methodology, J.H., X.L., and J.L.; investigation, J.T., F.L., J.H., and Y.Z.; writing – original draft, J.T. and F.L.; writing – review and editing, Y.F and Y.Z.; funding acquisition, Y.F. and J.L.; resources, J.T. and F.L.; supervision, Y.F.

## Declaration of interests

The authors declare no competing interests.

## STAR★Methods

### Key resources table

**Table:** 

REAGENT or RESOURCE	SOURCE	IDENTIFIER
**Antibodies**		

Rabbit monoclonal anti-PAR2	Abcam	Cat#ab180953; RRID:AB_3674622
Goat polycional anti-β-arrestin 2	Abcam	Cat#ab31294; RRID:AB_2060265
Mouse monoclonal anti-p-ERK	Santa cruz	Cat#sc-7383; RRID:AB_627545
Mouse monoclonal anti-ERK1/2	Santa cruz	Cat#sc-514302; RRID:AB_2571739
Rabbit polyclonal anti-IL-17	Affinity	Cat#DF6127; RRID:AB_2838094
Rabbit polyclonal anti-ZO-1	Thermo Fisher Scientific	Cat#61-7300; RRID:AB_138452
Mouse monoclonal anti-Occludin	Thermo Fisher Scientific	Cat#33-1500; RRID:AB_2533101
Mouse monoclonal anti-Claudin-1	Thermo Fisher Scientific	Cat#37-4900; RRID:AB_2533323
Mouse monoclonal anti-GAPDH	Proteintech	Cat#CL594-60004;RRID:AB_2919886
Alexa Fluor 555-labeled donkey anti-rabbit IgG	Beyotime	Cat#A0453; RRID:AB_2890132
Alexa Fluor 647-labeled Goat Anti-Mouse IgG	Beyotime	Cat#A0473; RRID:AB_2891322
Dylight 488-labeled donkey anti-goat IgG	Abcam	Cat#ab96935, RRID:AB_10679538
Rabbit anti-goat IgG-HRP	Santa cruz	Cat#sc-2768, RRID:AB_656964
Goat anti-mouse IgG-HRP	Santa cruz	Cat#sc-2005, RRID:AB_631736
Goat anti-rabbit IgG-HRP	Proteintech	Cat#SA00001-2, RRID:AB_2722564

**Bacterial and virus strains**		

*M. globose* type strain CBS9597	Luo et al.^17^	N/A
NMY51 yeast	LSM Biological	Cat#S0094

**Biological samples**		

Healthy adult skin tissue	This paper	N/A
Human MF lesions	This paper	N/A
Infected mouse skin tissue	This paper	N/A

**Chemicals, peptides, and recombinant proteins**		

PAR2 antagonist FSLLRY-NH2	Sigma-Aldrich	Cat#SML0714
PAR2 agonist SLIGRL-NH2	Sigma-Aldrich	Cat#S9317
Lipofectamine(RNAiMAX transfection reagent)	Thermo Fisher Scientific	Cat#13778150

**Critical commercial assays**		

TRIzol reagent	Thermo Fisher Scientific	Cat#12183018A
*Evo M-MLV* reverse transcription Kit	Aikori Biological	Cat#AG11705
SYBR Green *ProTaq* HS Pre-mixed qPCR Kit (with ROX)	Aikori Biological	Cat#AG11718
Duolink® *In Situ* red test reagent	Sigma-Aldrich	Cat#DUO92008
Duolink® In-situ PLA® probe anti-Rabbit PLUS	Sigma-Aldrich	Cat#DUO92002
Duolink® in-situ PLA® probe anti-mouse MINUS	Sigma-Aldrich	Cat#DUO92004
Duolink® in-situ PLA® detection mounting medium, containing DAPI	Sigma-Aldrich	Cat#DUO82040

**Deposited data**		

Proteomics data of MF	This paper	Science Data Bank: https://doi.org/10.57760/sciencedb.29934
Raw images	This study	Mendeley Data: https://data.mendeley.com/datasets/ffxppsr7p2/1

**Experimental models: Cell lines**		

HaCaT cells	iCell Bioscience Inc.	Cat# iCell-h066

**Experimental models: Organisms/strains**		

C57BL/6JGpt-*F2rl1*^*em6Cd3832*^/Gpt	Gempharmatech	Cat#T006568

**Oligonucleotides**		

Primer of PAR2 (Forward, 5′-GGCAC TCCAGGAAGAAGGCAAAC-3’; Reverse, 5′-CAGGGCAGGAATGAAGATGGTCTG-3′)	Sangon Biotech	N/A
Primer of β-arrestin 2 (Forward, 5′-TTTTGTTCTTATGCACCCC AAG-3’; Reverse, 5′-ATGTCAT CATCTGTGGCATAGT-3′)	Sangon Biotech	N/A
Primer of ERK1(Forward, 5′-TCTGCTACTTCCTCTAC CAGAT-3’; Reverse, 5′-CAGG CCGAAATCACAAATCTTA-3′)	Sangon Biotech	N/A
Primer of ERK2(Forward, 5′-ATGGTGTGCTCTGCTTA TGATA-3’; Reverse, 5′-TCTT TCATTTGCTCGATGGTTG-3′)	Sangon Biotech	N/A
Primer of IL-17(Forward, 5′-GAGATATCCCTCTGTG ATCTGG-3’; Reverse, 5′-GACA GAGTTCATGTGGTAGTCC-3′)	Sangon Biotech	N/A
Primer of ZO-1(Forward, 5′-ACATTGCCGCCAGCCAT CTC-3’; Reverse, 5′-ATCTAT CCACACCATCAGCTTCAGG-3′)	Sangon Biotech	N/A
Primer of Occludin (Forward, 5′-AACTTCGCCTGTGGATG ACTTC-3’; Reverse, 5′-TTTG ACCTTCCTGCTCTTCCCTTTG-3′)	Sangon Biotech	N/A
Primer of Claudin-1 (Forward, 5′-AGGTACGAATTTGGTCAGG CTCTC-3’; Reverse, 5′-GGGAC AGGAACAGCAAAGTAGGG-3′)	Sangon Biotech	N/A
Primer of GAPDH (Forward, 5′-TGCACCACCAACTGCT TAG-3’; Reverse, 5′-AGTAGA GGCAGGGATGATGTTC-3′)	Sangon Biotech	N/A
siRNA of PAR2 (Sense, 5′-CCAUGUACCUGAUCUGC UUTT-3’; Antisense, 5′-AAGC AGAUCAGGUACAUGGTT-3′)	GenePharma	N/A
siRNA of β-arrestin 2 (Sense, 5′-GACCGACUGCUGAAGA AGUTT-3’; Antisense, 5′-ACU UCUUCAGCAGUCGGUCTT-3′)	GenePharma	N/A
Scramble siRNA (Sense, 5′-UUCUCCGAACGUGUGA CGUTT-3’; Antisense, 5′-AC GUGACACGUUCGGAGAATT-3′)	GenePharma	N/A

**Recombinant DNA**		

pDHB1-PAR2 (TACAATCAA CTCCAAGCTGGCCGCTCT AGAATGCGGAGCCCCAG CGCGGCGTGGCTG;TGG CCGAGGCGGCCAAGAT ATACCATGGTCAATAGGA GGTCTTAACAGTGGTTGAAC)	Shengzhe Biotech	N/A
pPR3-N-β-arrestin 2 (CGGG ATCCGCATTGGCGCGGGG AGGA;AAAAGTACTTGCTT AGTAAAAGATTTATTTTCTTCTT)	Shengzhe Biotech	N/A
pPR3-N-ZO-1 (ATCCCGG GAAATGTCCGCCAGAGCT GCG;GAGGTCGACTTAAA AGTGGTCAATAAGGACAGA)	Shengzhe Biotech	N/A

**Software and algorithms**		

GraphPad prism(version 9.4.0)	GraphPad Software	RRID: SCR_002798
Image J	NIH	RRID: SCR_003070
Adobe Illustrator CS6	Adobe Inc.	RRID:SCR_010279

### Experimental model and study participant details

#### MF lesions and normal skin

Nine MF lesions and normal skin (CTRL) were collected from the Department of Dermatology, Affiliated Hospital of Guangdong Medical University. The study was approved by the Hospital’s Ethics Committee (No. PJKT2024-193). Inclusion criteria: (1) Typical manifestations of MF; (2) Calcofluor white stain of skin scrapings showing *Malassezia* spores and/or hyphae; (3) No systemic antifungal drugs, glucocorticoids or immunosuppressants within one month and no topical antifungal drugs within two weeks; (4) No congenital and acquired immunodeficiency.

#### Cell lines and culture conditions

HaCaT cells (cat#iCell-h066) were purchased from the iCell Bioscience Inc. (Shanghai, China) and cultured in high-glucose Dulbecco’s modified Eagle medium (Gibco, NY, USA) with 10% fetal bovine serum (Gibco) at 37°C in 5% CO_2_.The HaCaT cells have been authenticated by iCell Bioscience Inc., using a 21-STR amplification protocal. The cells were no mycoplasma contamination as tested by TEM.

#### Fungal culture

*M. globosa* type strain CBS9597 was obtained from the Institute of Dermatology, Chinese Academy of Medical Sciences and Peking Union Medical College (Nanjing, China).^17^ The yeasts were cultivated in modified Leeming-Notman broth for 4-5 days at 32°C, then harvested by centrifugation, washed thrice in PBS and diluted in medium.

#### Animals

*Par2*^*-/-*^ and wild-type (WT) C57BL/6 mice were purchased from GenePharma (Jiangsu, China). The identification profiles for *Par2*^*-/-*^ and WT mice are provided in Figure S4. Genotype mice were indentified using two pairs of primers (Primers ①: Forward, 5′-CTCAGTGGGAGGATGTTTATGAAG-3′, Reverse, 5′-CTGACCGTTCTATTCCAGAAATACAG-3′, Band size: WT: 4100 bp, *Par2*^*-/-*^: 268 bp;Primers ②: Forward, 5′-CTCAGTGGGAGGATGTTTATGAAG-3′, Reverse, 5′-CAACCACCAGCAGTCTTTGATTG-3′, Band size: WT: 380 bp, *Par2*^*-/-*^: 0 bp) (Figure S4). The PCR program was as follows: 95 °C for 5 min; 98 °C for 30 s, 65 °C for 30 s,72 °C for 45 s (20 cycles); 98 °C for 30 s, 55 °C for 30 s,72 °C for 45 s (20 cycles); 72 °C for 5 min, 10 °C hold. The animal experiments were approved by the Affiliated Hospital’s Ethics Committee of Guangdong Medical University (No. AHGDMU-LAC-Ⅱ(1)-2302-A055). Littermates of the same sex were randomly assigned to experimental groups, and the breeding was conducted in the SPF-grade animal breeding facility of Guangdong Medical University Affiliated Hospital. Previous multiple studies^9^^,^^74^^,^^75^ by our research group have demonstrated that males are more susceptible to MF than females, highlighting a gender difference. Consequently, subsequent studies used male mice as the infection model. Male mice aged 6-8 weeks were chosen. *M. globosa*-infected mouse skin models were established as previous reports.^17^^,^^18^ Mice were anesthetized by intraperitoneal injection of 1% pentobarbital sodium. Their dorsal skin was stripped once using 3M Durapore Surgical Tape (3M Health Care, MN, USA) after hair shaving, and then treated topically with 200 μL of 10^9^ spores/mL *M. globosa* or glycerol once daily for seven consecutive days.

### Method details

#### LC-MS/MS analysis

Three MF lesions and normal skin were collected from the Department of Dermatology, Affiliated Hospital of Guangdong Medical University. SDT (4%SDS, 100mM Tris-HCl, pH7.6) buffer was used for sample lysis and protein extraction. The amount of protein was quantified with the BCA Protein Assay Kit (Beyotime, Shanghai, China).The proteins were separated on SDS-PAGE gel (constant voltage 180V, 45 min). Protein digestion by trypsin was performed according to filter-aided sample preparation (FASP) procedure described by Matthias Mann.^76^ The digest peptides of each sample were desalted on C18 Cartridges (Sigma-Aldrich, MI, USA). LC-MS/MS analysis was performed on a timsTOF Pro mass spectrometry (Bruker, Karlsruhe, Germany) that was coupled to Evosep One system liquid chromatography (Evosep, Odense, Denmark). Finally, the MS data of samples were analyzed by bioinformatics as previous reports.^77^^,^^78^

#### Cell viability and ELISA

Cell viability was determined with the Cell Counting Kit-8 (CCK8, Beyotime, Shanghai, China), following the manufacturer’s protocol. HaCaT cells were plated in 96-well plates at a density of 3,000 cells per well and allowed to adhere for 24 h before treatment. Subsequently, 10 μL of CCK-8 solution was added to each well, and after 2 h of incubation, the absorbance at 450 nm was measured using a microplate reader. Interleukin-17 (IL-17) levels were quantified using commercial enzyme-linked immunosorbent assay (ELISA) kits (Meimian Industrial, Jiangsu, China) in strict adherence to the manufacturer’s instructions. Cell culture supernatants, tissue culture supernatants, and serially diluted standards were coated onto microplate wells. Following a washing step, the wells were blocked and then incubated with an anti-IL-17 primary antibody at 37°C for 2 h. After further washing, the wells were incubated with a horseradish peroxidase (HRP)-conjugated secondary antibody, washed again, and developed with substrate. The reaction was stopped, and absorbance at 450 nm was recorded using a microplate reader.

#### Reverse transcription quantitative PCR (RT-qPCR)

RT-qPCR was performed as previously described.^79^ Total RNA was extracted using TRIzol reagent (Thermo Fisher Scientific, USA) and reverse transcribed using *Evo M-MLV* reverse transcription Kit (Aikori Biological Engineering CO., Ltd, Changsha, China). mRNA expression was quantified via the SYBR green method (Aikori Biological Engineering CO., Ltd, Changsha, China) on a reverse transcription machine (LC480, USA). Specific primers of PAR2, β-arrestin 2, EKR1, ERK2, IL-17, ZO-1, Occludin, Claudin-1 and GAPDH were synthetized by Sangon Biotech (Shanghai, China). All samples were normalized to endogenous controls and fold changes calculated relative to control. The control GAPDH was used for normalization, and relative mRNA levels were calculated using the 2^-ΔΔCT^ method.

#### Western blotting analysis

The protein fractions were extracted using Protein Extraction Kit (Beyotime, Shanghai, China). Based on our previous experiment,^80^ the blots were incubated with primary antibodies PAR2, β-arrestin 2, p-ERK, ERK1/2, IL-17, ZO-1, Occludin, Claudin-1, and GAPDH at a 1:1000 dilution. They were then incubated with HRP-conjugated secondary antibodies (Proteintech, Wuhan, China) and visualized using enhanced chemiluminescence (Millipore, MA, USA). All experiments were conducted with three independent repetitions.

#### Immunohistochemical staining (IHC)

Immunostaining for PAR2, IL-17, ZO-1, Occludin, and Claudin-1 was performed on paraffin-embedded sections as previously described.^75^ Following antigen retrieval and blocking, paraffin-embedded sections were incubated with primary antibodies at 4°C overnight, after which they were treated with corresponding horseradish peroxidase (HRP)-conjugated secondary antibodies. Chromogenic detection was carried out using diaminobenzidine (DAB), and images were acquired using light microscopy.

#### Immunofluorescent staining (IF)

HaCaT cells were cultured in confocal Petri dishes and treated with 30 MOI *M. globosa* for 24 hours. Immunofluorescence staining for PAR2, β-arrestin 2, and ZO-1 was conducted as previously described.^80^ Specimens (tissues or cells) were fixed in 4% paraformaldehyde (Solarbio, Beijing, China) for 30 minutes, followed by permeabilization and blocking. The samples were then incubated with appropriate primary antibodies, followed by a light-protected incubation with fluorophore-conjugated secondary antibodies. Nuclear staining was performed using DAPI, and slips were mounted with an anti-fade medium. Image acquisition and observation were carried out on a laser scanning confocal microscope.

#### Periodic acid-Schiff staining (PAS)

The experimental protocol was conducted following the manufacturer’s instructions for the Periodic Acid Schiff (PAS) Stain Kit (Solarbio, Beijing, China). Paraffin-embedded tissue sections were deparaffinized in xylene and gradually rehydrated through a graded ethanol series. After oxidation in periodic acid solution, the sections were incubated with Schiff’s reagent at 30 °C for 45 min. Nuclei were counterstained with hematoxylin for 30 s, followed by differentiation in acid-alcohol and bluing in running tap water for 5 min. Finally, the sections were dehydrated, cleared, mounted, and imaged using light microscopy.

#### siRNA transfection

PAR2 and β-arrestin 2-targeted and scrambled siRNAs were designed by GenePharma (Shanghai, China). Briefly, adherent HaCaT cells were transfected with these siRNAs for 6-8 hours using Lipofectamin 3000 reagents and Opti-MEM (Thermo Fisher Scientific, CA, USA). Following transfection, the cells were cultured in fresh medium and co-cultured with 30 MOI *M. globosa* for 24 hours. The knockdown efficiency was verified by RT-qPCR and western blotting.

#### Transmission electron microscopy (TEM)

HaCaT cells were cultured on pre-sterilized Aclar film (Ted Pella, CA, USA) or 6-well cell cultrure plate(Nest, Jiangsu, China), treated according to the experimental settings. The samples were fixed with 3% glutaraldehyde and 1% osmium tetroxide, followed by gradient ethanol dehydration. They were then vacuum-infiltrated with ethanol or acetone resin using a microwave treatment instrument (Ted Pella, CA, USA) and polymerized in pure resin. After top buckle embedding, the blocks were trimmed and sectioned. The 70 nm ultrathin sections were double-stained with lead citrate and uranyl acetate, and observed at 80 kV on the JEM1400 (JEOL Ltd., Tokyo, Japan) for photography. The method is cited as previously described.^81^^,^^82^^,^^83^

#### Co-immunoprecipitation (Co-IP)

Cell lysates were extracted using Western and IP cell lysis buffer (Beyotime, Shanghai, China). Following centrifugation, the supernatants were incubated overnight with Protein A/G Magnetic Beads (MCE, Shanghai, China) and IP antibodies. The beads were then washed four times with the same lysis buffer, resuspended in 40 μL of 1% SDS-PAGE sample loading buffer, and boiled at 95°C for 5 minutes. The method is detailed as previously described.^84^^,^^85^^,^^86^

#### Proximity ligation assay (PLA)

HaCaT cells were cultured on sterile slides and prepared according to the experimental design. The PLA method is cited as previously described.^87^^,^^88^^,^^89^ The cells were fixed with 4% paraformaldehyde for 30 minutes, followed by treatment with Duolink® blocking solution (Sigma-Aldrich, MI, USA). Two species-specific primary antibodies were added and incubated at 4°C overnight. After incubation with Duolink® PLA probes, cells proceeded sequentially with Duolink® ligase for ligation and Duolink® polymerase for amplification. Nuclei were stained using DAPI-containing Duolink® *in situ* sealing agent (Sigma-Aldrich, MI, USA). Cells were then observed and analyzed with an LSM900 laser confocal microscope (ZEISS, Jena, Germany). A negative control without primary antibodies was included.

#### Membrane-based yeast two-hybrid (MbYTH)

After constructing the plasmid vector for the target gene using pDHB1 or pPR3-N (Figure S1 and Table S1), NMY51 yeast (LSM Bio, Wuhan, China) competent cells were prepared using LiAc method. Then, the plasmid vectors were transfected into the competent NMY51 yeast. The clones were successively screened using SD/-Leu/-Trp, SD/-Leu/-Trp/-His with 5 mM 3-AT, SD/-Leu/-Trp/-His/-Ade, and SD/-Leu/-Trp/-His/-Ade with 5 mM 3-AT. The continuously growing clones indicated the presence of protein interactions; otherwise, it would be a false positive result. The experimental groups were detailed in Table S1. The method reference remains as previously mentioned.^90^^,^^91^^,^^92^

### Quantification and statistical analysis

Statistical analyses were performed using GraphPad Prism software (version 9.4.0). Comparisons between two groups were made using unpaired two-tailed Student’s t-tests (Figures 1B, 1D, 6C, 6D, 6G, and 6H). For datasets containing one independent variable in multiple groups, a one-way ANOVA followed by Dunnet’s multiple comparisons test was performed (Figures 2G and S3C). For multiple group comparisons, a two-way ANOVA with Bonferroni’s (Figures 2A, S3B, and S3D), or Šídák’s (Figures 4B, 4D, 4E, 4G, 5B, 5D, 5F, and S3A) multiple comparisons test was applied. ∗*p* < 0.05, ∗∗*p* < 0.01, ∗∗∗*p* < 0.001, and ▲▲▲*p* < 0.001(Figure S3B) were considered statistically significant. A Pearson’s correlation analysis was conducted, and the values represent the correlation coefficient (Figures 2D and 2F).
