# Supplementary material for: PAR2-β-arrestin 2-ERK axis mediates Malassezia globosa-induced IL-17 response by disrupting ZO-1 in keratinocytes
Source: iScience. 2026 Apr 8;29(5):115646. doi: 10.1016/j.isci.2026.115646 (PMC13127400; doi:10.1016/j.isci.2026.115646)
Supplement: Document S1. Figures S1–S4 and Table S1 [file mmc1.pdf]

iScience, Volume *29*

Supplemental information

PAR2-β-arrestin 2-ERK axis mediates *Malassezia globosa*-induced IL-17 response by disrupting ZO-1 in keratinocytes

Jinfeng Tang, Fanggu Li, Jiaqing Hong, Xueying Li, Ying Zhou, Jie Liu, and Yiming Fan

Table S1. Experimental groupings in MbYTH. Relative to STAR Methods.

	a	b	c	d	e
Groups	Positive control group	Negative control group	Experimental group	Self-activating group 1	Self-activating group 2
PAR2/control NR1I2	pTSU2-APP	pTSU2-APP	pDHB1-PAR2	pDHB1-PAR2	pDHB1
	pNubG-Fe65	pPR3-N	pPR3-N-NR112	pPR3-N	pPR3-N-NR112
PAR2/ZO-1	pTSU2-APP	pTSU2-APP	pDHB1-PAR2	pDHB1-PAR2	pDHB1
	pNubG-Fe65	pPR3-N	pPR3-N-ZO-1	pPR3-N	pPR3-N-ZO-1
PAR2/β-arrestin 2	pTSU2-APP	pTSU2-APP	pDHB1-PAR2	pDHB1	
	pNubG-Fe65	pPR3-N	pPR3-N-β-arrestin 2	pPR3-N-β-arrestin 2	
β-arrestin 2/ZO-1	pTSU2-APP	pTSU2-APP	pDHB1-β-arrestin 2	pDHB1-β-arrestin 2	pDHB1
	pNubG-Fe65	pPR3-N	pPR3-N-ZO-1	pPR3-N	pPR3-N-ZO-1

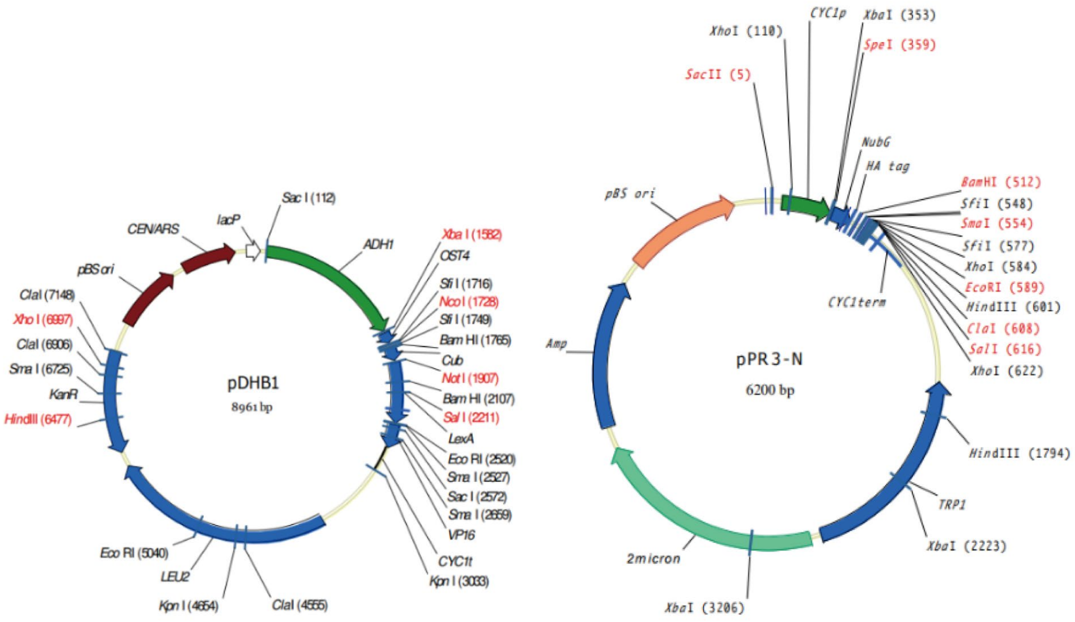

Figure S1. Mapping of pDHB1 and pPR3-N vectors. Relative to STAR Methods.

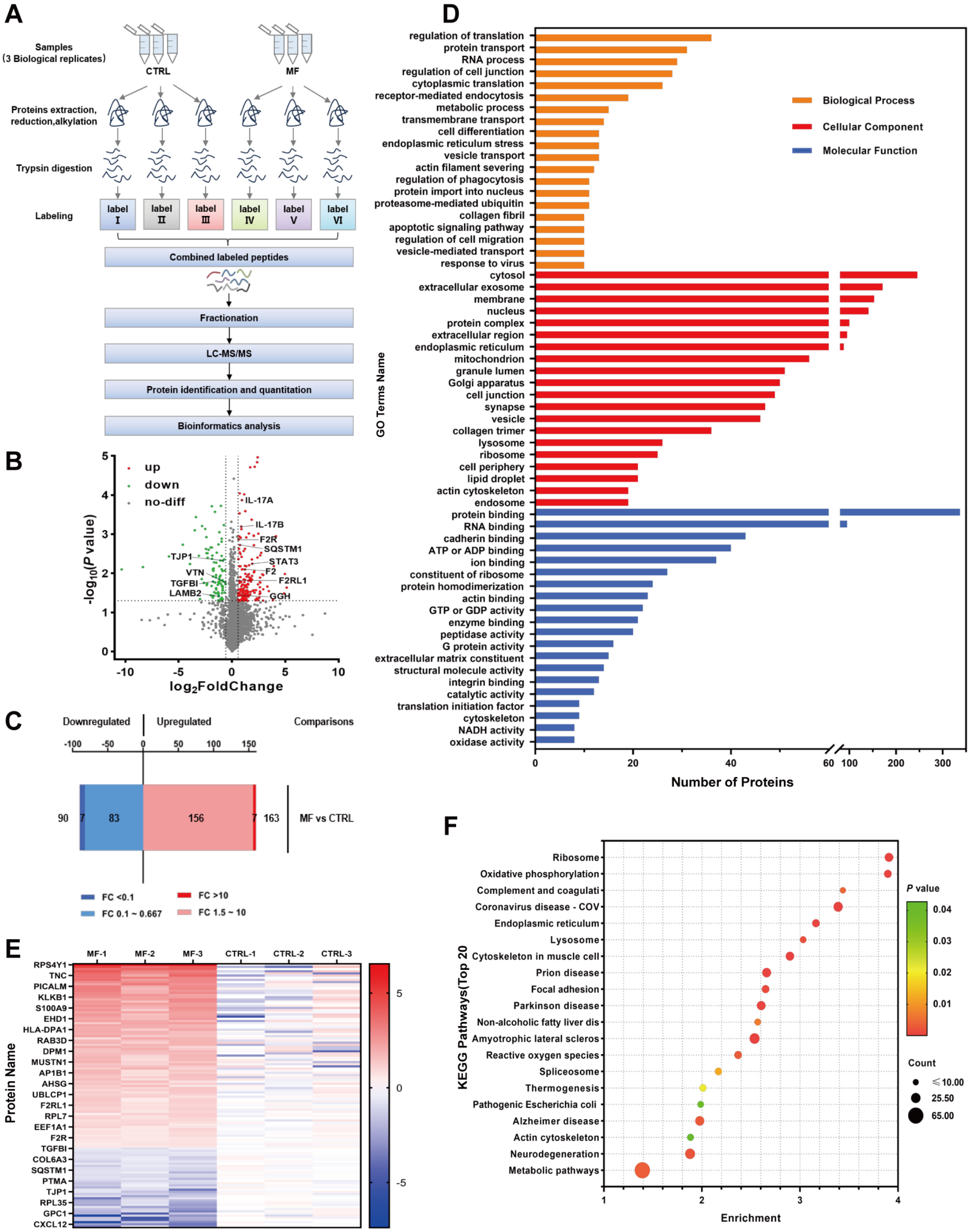

Figure S2. Analysis of proteomics in MF lesions and CTRL. Relative to Figure 1.

(A) Workflow diagram of LC-MS/MS analysis.

(B) Volcano plot of proteomics. Red points indicated significantly up-regulated DEPs and blue points indicated significantly down-regulated DEPs. Gray dots represented non-DEPs.

(C) Histogram of significantly DEPs. Dark blue for >10 folds down-regulation, light blue for

1.5-10 folds down-regulation, pink for 1.5-10 folds up-regulation, and red for >10 folds up-regulation.

(D) GO annotation statistical map of DEPs. Y-axis represented GO secondary function annotation information, including biological process, cellular component and molecular function, distinguished by orange, red, and blue, respectively. X-axis represented the number of DEPs in each functional category.

(E) Cluster analysis results of DEPs. Depicted as a tree heat map, with each column representing a sample group and each row representing a protein. Red indicated significantly up-regulated proteins, blue represents significantly down-regulated proteins, and white represents no protein quantitative information.

(F) Bubble diagram of KEGG pathway enrichment. X-axis represented the enrichment factor and Y-axis the statistical results of DEPs under each KEGG pathway. The bubble colors indicated the significance of enriched KEGG pathways, with colors closer to red representing smaller *p*-value, corresponding to higher significance levels of metabolic pathway enrichment.

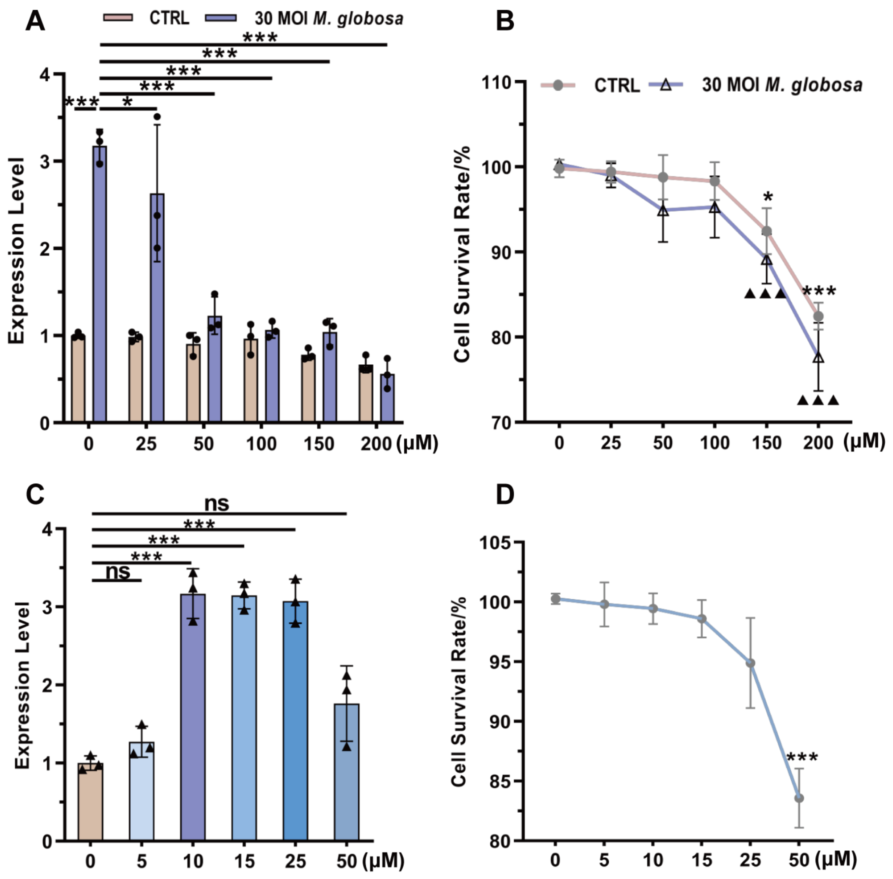

Figure S3. Concentrations screening of PAR2 antagonist and agonist. Relative to Figure 4.

(A, B) *PAR2* mRNA level and Cell survival rate in 30 MOI *M.globosa* infection of HaCaT cells pretreated with FSLLRY-NH2 by qRT-PCR and CCK8 test.

(C, D) *PAR2* mRNA level and Cell survival rate in HaCaT cells pretreated with SLIGRL-NH2 by qRT-PCR and CCK8 test. These experiments were all conducted with 3 independent trials. Values are means ± SD, compared with CTRL (0 μM FSSLRY-NH2 or SLIGRL-NH2), * $p < 0.05$, *** $p < 0.001$; compared with 30 MOI *M.globosa* infection (0 μM FSSLRY-NH2), ▲▲▲ $p < 0.001$.

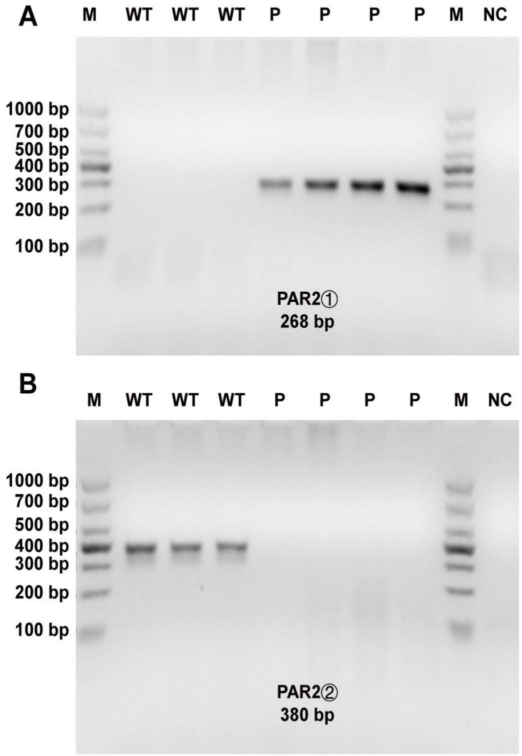

Figure S4. Identification of *Par2*$^{-/-}$ and WT mice from littermates. Relative to STAR Methods.

(A) Screening mice with PAR2 primers① (Band size: WT: 4100 bp, *Par2*$^{-/-}$: 268 bp) by PCR test.

(B) Screening mice with PAR2 primers② (Band size: WT: 380 bp, *Par2*$^{-/-}$: 0 bp) by PCR test. M: 1000 bp DNA marker; WT: WT mouse; P: *Par2*$^{-/-}$ mouse; C: negative control.
